# *‘The mosquitoes are preparing to attack us’*: knowledge and perceptions of communities in south-eastern Tanzania regarding mosquito swarms

**DOI:** 10.1186/s12936-019-2686-1

**Published:** 2019-02-26

**Authors:** Marceline F. Finda, Emmanuel W. Kaindoa, Anna P. Nyoni, Fredros O. Okumu

**Affiliations:** 1 Environmental Health and Ecological Science Department, Ifakara Health Institute, P. O. Box 53, Ifakara, Tanzania; 2 School of Public Health, Faculty of Health Sciences, University of the Witwatersrand, Johannesburg, South Africa; 3 Institute of Biodiversity, Animal Health and Comparative Medicine, University of Glasgow, Glasgow, G12 8QQ UK

**Keywords:** Malaria transmission, Mosquito mating, Male mosquitoes, Mosquito swarms

## Abstract

**Background:**

Targeting swarms of male *Anopheles* mosquitoes with techniques such as aerosol spraying could potentially suppress malaria vector populations and parasite transmission. Unfortunately, research on *Anopheles* swarming behaviours is limited, particularly in East Africa where only a handful of studies have been done. New evidence has recently emerged that such swarms are common even in Tanzania, where they could be readily identified and characterized by community-based volunteers, and potentially targeted for control. However, improved understanding of public knowledge and perceptions will be crucial for successful uptake of any interventions targeting swarms.

**Methods:**

Explanatory sequential mixed methods approach was used to assess knowledge and perceptions regarding mosquito swarms among community members in Ulanga and Kilombero districts, south-eastern Tanzania. A survey questionnaire was administered to 307 respondents, after which focus group discussions were conducted to clarify responses regarding mosquito swarms and malaria transmission. Findings from both study components were used to draw qualitative inferences.

**Results:**

Most community members (83%) had previously seen mosquito swarms, predominantly in farms, over long grasses or bushes, above ponds and over roofs of houses and pit-latrines. However, there was little evidence that community members could distinguish between mosquito swarms and those of other insects. Neither were they aware that swarms consisted mostly of male mosquitoes. Swarming was associated with mosquitoes preparing to attack people, foraging for food, playing or resting. Very few respondents associated swarming with mosquito mating. Nearly all community members were willing to accept interventions targeting mosquito swarms; and approximately three quarters would pay for such interventions, between 0.9 and 2.3 USD/year.

**Conclusion:**

Majority of the community members recognized presence of mosquito swarms in their communities but did not associate these swarms with mosquito mating. Instead, swarming was associated with mosquitoes seeking food or planning to attack people, and thus were generally considered dangerous. This understanding created the basis for wide-acceptance of interventions targeting swarming mosquitoes. Although the likelihood of actual interventions targeting swarms is still low, such community knowledge will be crucial in future field studies of mosquito swarms and possible inclusion of community members in mosquito control efforts.

## Background

Although malaria cases and mortality have dramatically reduced over the past decade [1–3], the disease continues to be a major public health problem, predominantly in sub-Saharan Africa. As of 2017, a total of 219 million cases and 435,000 deaths were reported, over 90% of which were in WHO Africa region and two-thirds of which occurred among children under 5 years [1]. Vector control interventions, mainly long-lasting insecticidal nets (LLINs) and indoor residual spraying (IRS) have hugely contributed to the recent reductions in malaria burden [1–3], but their efficacy is rapidly reaching its limits, mainly due to resistance against public health pesticides, as well as behavioural resistance observed in mosquitoes [4–6]. Moreover, these interventions offer protection mainly when people are resting or sleeping indoors, leaving them vulnerable when outdoors [4, 7, 8]. These limitations have made malaria elimination challenging; as transmission continues to persist, though at lower levels, even in communities with high LLIN coverage [8, 9]. To make progress in ‘shrinking the malaria map’ it is therefore necessary to consider alternative complementary interventions that can target the persistent transmission driven by mosquitoes that currently escape LLINs and IRS. A thorough re-examination of the overall ecology of the malaria vectors [10] is ever more pressing, including not only the blood-feeding and resting habits commonly targeted by LLINs and IRS, but also other mosquito habits indoors and outdoors. These may include the oviposition-site seeking, sugar-feeding, mating and resting behaviours among others.

Mating behaviour is one of the most important, yet one of the least studied aspects of mosquito biology [11]. Malaria mosquitoes, like most other arthropods, commonly mate in swarms, which occur at specific times and places and include mosquitoes of the same species, making this a potential alternative for targeting adult vector populations [11–18]. Improved understanding of mosquito swarms could therefore potentially provide new opportunities for expanding vector control options. Indeed, the concept of targeting swarming mosquitoes in control of malaria vectors has already proven efficacious in reducing *Anopheles* mosquitoes in Burkina Faso [16], where targeting swarming mosquitoes with a mixture of carbamate and pyrethroid aerosol resulted in over 80% decrease vector population. Additional studies are currently underway in both Burkina Faso and Tanzania to demonstrate the potential of targeting *Anopheles* mosquito swarms for control. If successful, this would open up new opportunities for improved control of malaria transmission.

Unfortunately, studies on the male *Anopheles* swarming behaviour have been minimal, particularly in East Africa, where only a handful of studies have been completed in the past [15]. In Tanzania for example, there were no reports of *Anopheles* swarms for more than 30 years, since the one study by Marchand in 1983, which reported presence of *Anopheles* swarms in northern Tanzania [15]. More recently however, Kaindoa et al., conducted an exploratory study in south-eastern Tanzania with help from the local community volunteers and identified 216 *Anopheles* swarms consisting of between 20 and 360 mosquitoes each. Nearly all (99%) of all the mosquitoes in the swarms were *Anopheles gambiae* sensu lato (s.l.) males, with a very small number of females. The swarms appeared after sunset almost always at the same locations, and lasted 15 to 25 min. Swarm markers included rice fields, bare ground, termite mounds, banana trees, trash heaps, and brick piles [18]. The team in Tanzania comprehensively characterized these swarms and described some potential implications of the new discoveries, in malaria control. One potential consideration was that since the swarms occur at exactly the same locations and same times every day, they could be easily targeted by interventions against male mosquitoes. Achieving this would however require active mapping and comprehensive characterization of the swarms.

Whereas past sociological studies in rural south-eastern Tanzania have demonstrated high level of awareness on the role of mosquitoes in malaria transmission and the different options for control [19–21], it is reasonable to assume a much lower level of awareness of interventions targeting male mosquitoes. This is partly because current interventions do not typically target male mosquitoes, and the overriding narrative in public health community is that female mosquitoes are the important ones in transmission. Indeed, in surveys where people are asked to identify how they would control malaria mosquitoes, the theme of male mosquitoes rarely features [22, 23]. This is also observed when experts are interviewed on vector control programmes [24, 25]. Given the current situation, it is essential that any plans to eventually roll out interventions targeting male mosquitoes should first assess knowledge and perceptions of the communities, on subjects such as male mosquitoes, mosquito swarms and whether targeting male mosquitoes could interrupt transmission.

The aim of this study was therefore to assess the knowledge and perception of community members regarding the presence and role of *Anopheles* mating swarms and male mosquitoes in malaria transmission, and the need and acceptance levels of swarm-targeting interventions for the control of malaria vectors and transmission.

## Methods

### Study area

**Fig. 1:**
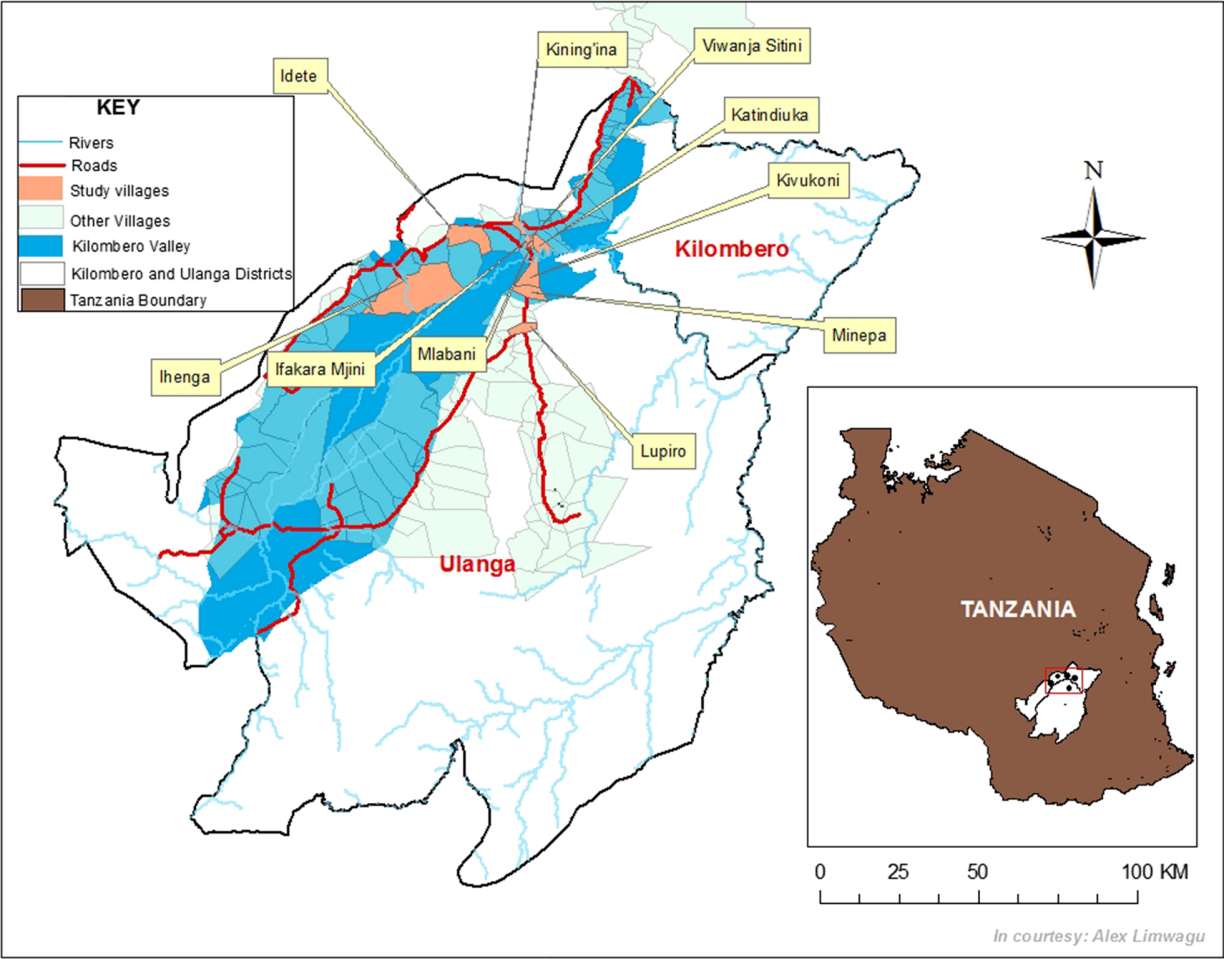
Map of the villages in Ulanga and Kilombero districts, south-eastern Tanzania, in which this study was conducted (Map prepared by Alex Limwagu, Ifakara Health Institute)

This study was done in Ulanga and Kilombero districts in the Kilombero Valley in south-eastern Tanzania (Fig. 1). In Ulanga district, the study was done in Kivukoni, Lupiro and Minepa villages, the same villages in which Kaindoa et al. initially identified *Anopheles* swarms [18]. In urban settings of the Kilombero district, the study was done in Ifakara town and its surrounding sub-villages including Ifakara mjini, Katindiuka, Mlabani and Viwanja sitini. Lastly, in the rural settings of the Kilombero district the study was done in Kining’ina, Idete, and Ihenga villages (Fig. 1). Malaria transmission in the valley is perennially moderate and there is a high mosquito density throughout the year, peaking between March and May [26–28]. The major mosquito control intervention is LLINs, which are universally distributed by the government every 3–4 years [29]. The last mass distribution was done between June and October 2016. Inhabitants are mostly seasonal rice farmers though there is also irrigated rice farming done during the rest of the year.

A detailed description of this area has been provided in Kaindoa et al. [18, 26], Finda et al. [28] and Matowo et al. [6]. The most abundant malaria vector here is *Anopheles arabiensis*, but a recent study has shown that *Anopheles funestus*, which occurs in far lower densities is now responsible for most of the remaining malaria transmission in the area [26, 28]. There are also mosquitoes of genera, *Culex*, *Aedes* and *Mansonia*, which are mostly nuisance-biting, but co-occur with the *Anopheles* in the area. Evidence from past sociological studies suggest strong association by community members between mosquito densities and malaria transmission [19, 23], but no evidence exists of people’s ability to distinguish between mosquito species or sexes.

### Study design and data collection

**Fig. 2:**
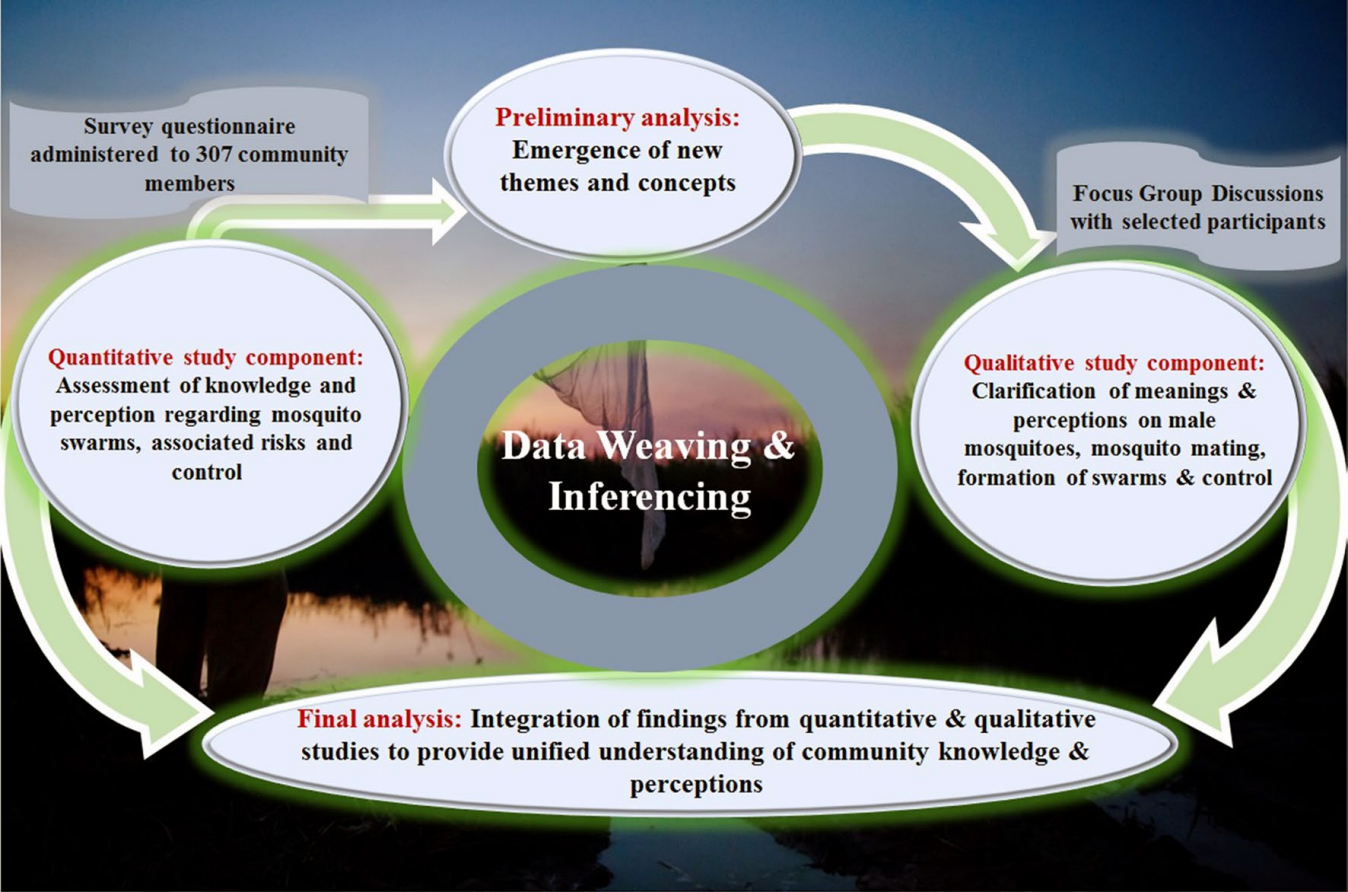
Illustration of the explanatory sequential mixed methods approach used to examine community knowledge and perceptions of *Anopheles* mosquito swarms and associated risks. This approach had two strands, the first being a quantitative survey of 307 households, and the second arm being Focus Group Discussions with selected members. New themes and concepts were generated during the first stage analysis, after which there was data weaving and inferencing using information from the two strands to generate a comprehensive understanding from the perspective of the respondents

Explanatory sequential mixed methods approach [30, 31] was used in this study (Fig. 2). A quantitative component was conducted first, which involved a structured questionnaire survey to assess knowledge and perceptions regarding mosquitoes, mosquito swarms and their role in malaria transmission. Preliminary analysis of the survey findings was followed by a qualitative component, which involved a series of focus group discussions to clarify some of the responses from the initial survey. Findings from the two components were used to make inferences (Fig. 2).

### Quantitative component

Using the pre-existing household data from the original Ifakara Health and Demographic Surveillance System (IHDSS) [32] and help from community leaders, 307 households were randomly selected, about evenly distributed across the 10 study villages (Fig. 1). A structured questionnaire was administered to one representative from each of the 307 households. Concepts investigated included participants’ perception of malaria risk and role of mosquitoes in transmission. Attitudes and practices relevant to malaria prevention and treatment were also assessed. Core components of the survey included participants’ knowledge and perceptions regarding mosquito swarms, what they associated the mosquito swarms with, and their willingness to accept any interventions that would target the swarms (Table 1).

**Table 1:** Description of the main themes and concepts investigated in the survey to assess knowledge and perceptions on mosquitoes, mosquito swarms and malaria transmission

	Concepts investigated	Specific questions asked by the interviewer	Relevance of the concepts
1	Perception of risks associated with mosquitoes	How big is the mosquito problem in your home/village?	Assessment of knowledge and perception of risk and vulnerability towards mosquitoes and malaria
		What diseases are transmitted by mosquitoes?	
		Have you or anyone in your family gotten malaria over the past 12 months?	
2	Burden of malaria, the interventions currently being used and costs incurred by households	How do you protect yourself or your family from mosquito bites?	Estimation of burden of malaria and the cost of treatment and prevention incurred by households
		How much did you spend for protecting against mosquito bites over the past 12 months?	
		How many members of your household have gotten malaria over the past 12 months?	
		How much did you spend on malaria treatment over the past 12 months	
3	Knowledge and perception about mosquito swarms and perceived risks associated with the swarms	Have you ever seen mosquito swarms (the interviewer described using gestures what he/she meant by swarms)?	Assessment of knowledge, awareness and perceptions regarding mosquito swarms and any risks associated with swarms
		What places did you see mosquito swarms?	
		What time of the day did you see mosquito swarms?	
		What do you think mosquitoes do when they swarm?	
		Do you think swarming mosquitoes are dangerous?	
4	Acceptance and willingness to pay for interventions that target mosquito swarms	Do you think mosquito swarms should be prevented?	Assessment of the need for swarm-targeting interventions and willingness to use and contribute towards associated costs
		Would you be willing to use an intervention that would kill mosquito swarms?	
		Would you be willing to make financial contributions towards these interventions	
		How much would you be willing to pay annually for an intervention that effectively kills mosquito swarms?	

This survey was administered to 307 community members in Ulanga and Kilombero districts, Tanzania

### Qualitative component

To clarify the findings from the quantitative component, focus group discussions (FGDs) were done with a subset of the respondents of the survey. The discussions provided in-depth information on the community members’ understanding and perceptions of mosquito mating behaviours, breeding habitats and swarms. The discussions were semi-structured; the facilitator introduced a concept and the group discussed their knowledge and perceptions. Male and female participants were separated during the discussions in order to maximize participation. The discussions were conducted at the local village offices or in a classroom at the local primary schools. The discussions were done in Swahili, a local language in Tanzania, and were audio-recorded.

### Data processing and analysis

The data from the field surveys was checked, cleaned and coded. All questions on knowledge and perceptions regarding malaria, mosquitoes and mosquito swarms were scored as 1 or 0 to refer to correct and incorrect responses based on expert entomologists’ knowledge. Yes and No answers were also coded as 1 or 2 respectively. All descriptive data was summarized and presented as proportions.

The FGD verbatim were transcribed and translated to English, and notes taken during the discussions were incorporated into the written transcripts. The transcripts were then imported into NVIVO 12 Plus software [33] for coding. Deductive and inductive coding was used to extract themes. Preliminary findings from the initial survey were used to generate an FGD guide, which was used to develop deductive or topic codes, but other codes were also generated inductively based on detailed studying of the transcripts. Similar codes were grouped together and themes extracted from the patterns that emerged. Five FGDs were conducted, each with 6–8 participants ranging from 19 to 56 years of age. Points discussed included: (1) mechanisms mosquitoes use to transmit malaria, (2) characteristics of mosquito habitats, (3) main foods of mosquitoes, (4) role of male mosquitoes in disease transmission and (5) formation and characteristics of mosquito swarms and risks associated with the swarms.

Findings were presented using the integration principles and practices in mixed methods designs as described by Fetters et al, [34]. Weaving approach was used, in which both qualitative and quantitative findings were reported together based on the themes as illustrated in Fig. 2. Quantitative findings from the survey were presented, and explanations for some of the concepts were given from the FGDs. Direct quotations from the FGD participants were reported in some selected cases to further describe the themes.

## Results

### Socio-demographic characteristics of the respondents

Summary of socio-demographic information for the respondents is presented in Table 2. Sixty percent of all the respondents were females, and over 90% practiced subsistence farming. Nearly three quarters of the respondents had a primary school education, and 74.1% had a monthly household income of less than 200,000 Tanzanian Shillings (~ 90 USD/month). Average household size was 5.7 people ranging from 1 to 18 people per household; households occasionally included close family members and relatives living together. Every household visited had at least one mosquito bed net; there was an average of 2.8 bed nets per household, thus an average of ~ 2 people/net. Majority of the households (86.4%) had bricks or cement walls and corrugated iron roofs. Most households had at least one radio, a cellphone, a bicycle and at least one acre of farming land (Table 2).

**Table 2:** Socio-demographic characteristics of the survey respondents in the study districts of Ulanga and Kilombero, south-eastern Tanzania (n = 307)

Variables assessed	Category	Percentage (N)
Sex	Males	39.7 (122)
	Females	60.3 (185)
Level of education	No formal education	8.4 (26)
	Primary school	73.1 (224)
	Secondary school	17.2 (53)
	College/university	1.3 (4)
Primary occupation	No formal work	7.8 (24)
	Farmer (subsistence farming)	92.2 (283)
Average monthly household income	Less than 100,000 TZS (< 45 USD)	62.4 (191)
	100,000–200,000 TZS (45–90 USD)	11.7 (36)
	More than 200,000 (> 90 USD)	15.7 (48)
	Do not know/do not wish to disclose	10.2 (32)
Household assets	Farming land	87.1 (267)
	At least one cellphone	80.2 (246)
	At least one radio	59.1 (181)
	At least one bicycle	65.9 (202)
	Livestock (e.g. chicken, goats, cows)	87.3 (268)
	At least one television	20.5 (63)
Material for wall construction	Bricks and cement	86.4 (265)
	Mud and wood	13.6 (42)
Material for roof construction	Corrugated iron	81.9 (251)
	Thatch	18.1 (56)

### Knowledge and perceptions of the community members on the risk and burden of malaria

Nearly all respondents (96%) said that they had previously been infected with malaria. Three quarters of the respondents reported having had at least one case of malaria over the past 12 months in their household; there was an average of 2.3 cases for the past 12 months per household. Two-thirds of the respondents reported visiting health care facilities first to test for malaria before going to drug stores to get medications, but one-third reported going straight to the drug stores to get malaria medication whenever they felt malaria-like symptoms. Malaria symptoms commonly listed were head and body aches, chills and fevers, nausea or vomiting and fatigue. Others were diarrhoea, bitter mouth taste and flu-like symptoms.

The respondents reported having spent an average of 20,986 Tanzanian shillings (~ 9.5 USD) over the past year on malaria treatment. All of the respondents mentioned that bed nets were their main method for preventing mosquito bites and malaria transmission. Besides, all the respondents had received at least one LLIN through the government-backed universal coverage campaigns. Nearly two-thirds of the respondents had also purchased at least one additional net over the past 12 months, the average cost for such purchases being 14,602 Tanzanian Shillings (6.6 USD).

### Knowledge of community members on role of mosquitoes in malaria transmission

All 307 respondents said that mosquitoes were a big problem both in their homes and in their villages. The participants reported that mosquito density was high throughout the year, but peaked during the rainy season. Breeding habitats mentioned included standing water, dirty water, long grass and bushes. Dirty water was described as water that had been sitting in the open for a few days, giving mosquitoes time to lay eggs, and the eggs to mature into adult mosquitoes as this participant said, “*Mosquitoes are like house flies; they like to breed in dirty environment. I do not think mosquitoes can breed in clean water, they wait until the water is too dirty, you know when it has changed color, that is where they like to breed.”* (Female, 30). The surge of mosquitoes following rains was believed to be caused by increased water, long grass and generally dirtier environment: *“Mosquitoes breed a lot more during this rainy season because during this season the environment is not very clean. Because of the rain there is a lot water, long grass and trash. People do not burn their trash because it is always raining, so generally the environment is not clean and mosquitoes like that”* (Female 24).

Malaria-transmitting mosquitoes were believed to be mostly active after midnight (a time known in local language as ‘*usiku wa manane*’), when people were in deep sleep, hence considered a convenient time for mosquitoes to transmit malaria as this participant said, “*These mosquitoes start transmitting malaria at 2.00 a.m., but they can come as early as 1.00 a.m. It has to be late at night when everyone is asleep and all is quiet. They are called Anopheles mosquitoes, and they only come late in the night. When these mosquitoes are biting you, they pass malaria parasites at the same time”* (Female, 43).

### Male mosquitoes and malaria transmission: community understanding and perceptions on role of male mosquitoes in malaria transmission

Male mosquitoes were believed by some participants to be the nuisance mosquitoes that came earlier in the evening. While they were believed to feed on blood and water like their female counterparts, male mosquitoes were not believed to transmit malaria. Their bites were said to be different from those of female mosquitoes. According to some participants the bites of male mosquitoes were more intense, itchier and with bigger and more painful bumps compared to the female mosquito bites. On the contrary, female mosquitoes were believed to be cunning and that their bites were stealthy and mostly undetectable by the victims, which made them effective malaria vectors: *“Male mosquitoes do not transmit malaria. They are the ones that come out earlier in the evening, or during the day. They just bite, and their bite is very itchy and gives too many bumps, but they do not transmit malaria. Malaria is transmitted by female mosquitoes that come later in the night”* (Male, 50). There was also a view that male mosquitoes have a responsibility to ensure that their female counterparts successfully transmit malaria parasites to humans as this participant said, *“Male mosquitoes go with their women, they escort them to get the food, and they also get their food on the way. Then they also make sure that the female mosquitoes have spread the malaria parasites”* (Female, 43).

### Mosquito mating: knowledge and perceptions of the community members on mosquito mating behaviour

Mosquitoes were believed to mate in dark places at night, and mating was believed to be initiated by female mosquitoes when they were “on heat” as this participant said, “*I think it is the female mosquito that initiates the marriage deed* [euphemism for sex]*. When she feels like it is time to do it then she goes to look for a male and they do it. And they meet in the same places they hide. So they meet, do their business and have their babies there in the dark”* (Male, 34). Though uncommon, there were a few people with views that mosquitoes mate when they come out of their hiding places in the evening, when swarming. They were compared termite swarms seen following rains as this participant said, *“Mosquitoes are just like termites; during the rainy season in the evenings you see a lot of termites flying around, then after a few minutes you see pairs of termites following each other. Sometimes you can even see them do the marriage deed; you will see one on top of the other. Now I have never seen mosquitoes do this, but I imagine that they do it just like the termites do”* (Female, 45). Yet there were also views that mosquitoes do not mate due to their small size. Female mosquitoes were believed to lay their eggs in the water, after which males went to fertilize those eggs in the water as this participant said, *“I agree that mosquitoes do not do the marriage deed. They just lay their eggs. I think female mosquitoes are born with their eggs and lay them in the water, and then male mosquitoes look for those eggs and fertilize them. But I do not think that mosquitoes mate”* (Male, 31).

### Mosquito swarms: knowledge and perceptions of community members on formation and importance of mosquito swarms

Nearly three quarters of the survey respondents had seen mosquito swarms, most of these observations having been above water ponds or river streams, above bushes or long grass, under tree branches, above roofs of houses or pit latrines, and in the farms (Table 3). Other places listed included inside human houses, near light bulbs and over people’s heads. Majority of the respondents that had seen swarms (62.2%) reported seeing them after sunset, but some also mentioned seeing them during early morning hours (17.0%), during the day time (7.8%) and at night (23.5%). Only 8 of the 307 survey respondents associated mosquito swarms with mating, which is the main activity that medical entomology experts commonly associate with the swarms (Table 3). Some of the popular views were that when swarming, mosquitoes were either preparing to attack people, foraging for food and water or resting and playing as this participant said, *“I have seen them* [swarms] *after the sunset. I normally see them under a tree branch near my house. They are there every day, they never miss. I think they are normally making plans for the night, or they could be just resting. It is hard to know what they are really doing since we cannot ask them”* (Male, 32). Mosquito swarms were believed to be comprised of equal numbers of males and females meeting as it got darker, and were believed to also mate as this participant said, *“I think that they also do the marriage deed when they gather in the evening. That is when males and females meet, so they do it there”* (Female, 30). While mosquitoes in the swarms were believed to bite and even transmit diseases, hence dangerous (Table 3), mosquitoes were not deemed dangerous as they swarmed. Instead, they were believed to attack people after they had left the swarms as this participant said, *“I think when they are just gathering there they are harmless, but you know that they will come to bite you later, so they will still be dangerous, just not at that specific moment”* (Male, 23). Nevertheless, nearly all respondents of the survey and participants of the FGDs participants believed that mosquito swarms should be targeted to prevent the mosquitoes from attacking people later on.

**Table 3:** Knowledge and perceptions of community members about swarming mosquitoes

Variables assessed	Category	Percentage (N)
Whether participants had ever seen a mosquito swarm (n = 307)	Yes (Ever seen a swarm)	74.9 (230)
	No (Never seen a swarm)	25.1 (77)
Time of day when swarms were seen (n = 230)	Early morning hours	14.3 (33)
	During the day	7.8 (18)
	Around sunset hours	59.6 (137)
	During night hours	26.1 (60)
Places where swarms had been seen (n = 230)	On top of houses and toilets	41.3 (95)
	Over rice fields, long grass and bushes	29.6 (68)
	Over water ponds	13.9 (32)
	Near light bulbs	4.8 (11)
	Over people’s heads	7.8 (18)
	Inside human houses & toilets	2.6 (6)
Whether people knew reasons mosquitoes swarm (n = 307)	Know	20.2 (62)
	Do not know	79.8 (245)
What participants thought were reasons mosquitoes swarm (n = 62)	Planning an attack on humans	33.9 (21)
	Looking for food or water	32.2 (20)
	Playing or resting	21.0 (13)
	Mating	12.9 (8)
Effects of swarming mosquitoes (n = 187)	Transmit malaria and other diseases	67.9 (127)
	Nuisance biting/get into eyes	25.7 (48)
	Reproducing and increasing in number	6.4 (12)

### Interventions against mosquito swarms: perceptions and opinions of community members on the need to target mosquito swarms

Majority of the survey respondents (83%) said that it was important to kill swarming mosquitoes (Table 4), and listed interventions such as insecticide-sprays, cleaning the environment, mosquito traps and switching lights off. However, there was some skepticism about feasibility of killing swarming mosquitoes as this participant said, *“When mosquitoes are out in the open, it is just very hard to get them. Whatever you try to do, they will fly away and it is not easy to get them back. So, I do not know if it can be possible to kill a lot of them when they are out in the air.”* (Male, 34). Nearly all respondents (97.4%) of the survey said they would be willing to use an intervention that could kill mosquitoes while they are swarming. Majority (72.0%) said that they would be willing to pay between 2000 and 5000 Tanzanian Shillings (0.9–2.3 USD) per year while 9.4% said they would pay between 6000 and 10,000 Tanzanian Shillings (2.7–4.5 USD) yearly. The rest said that they would either not pay for malaria control intervention or they would wait to see how effective such interventions are.

**Table 4:** Perceptions and opinions of community members and their willingness to use or pay for interventions targeting swarms

Variables assessed	Category	Percentage (n)
Whether respondents thought swarms should be prevented (n = 307)	Yes	83.1 (255)
	No	16.9 (52)
Whether respondents thought swarms can actually be prevented (n = 307)	Yes	61.9 (190)
	No/don’t know	39.1 (117)
Methods community members suggested for preventing swarms (n = 190)	Insecticide-sprays	52.0 (99)
	Cleaning environment	34.7 (66)
	Using mosquito traps	13.3 (25)
Willingness by respondents to use special techniques for killing mosquito swarms (n = 307)	Yes	97.4 (299)
	No	2.6 (8)
Amount that respondents are willing to pay for swarm-targeting interventions per year (n = 299)	0	9.7 (29)
	2000–5000 (0.9–2.3 USD)	73.9 (221)
	6000–10,000 (2.7–4.5 USD)	9.7 (29)
	Depends on proven effectiveness	6.7 (20)

## Discussion

Targeting male mosquito mating behaviour has recently been shown to be effective in mass reduction of *An. gambiae* s.l. populations in Burkina Faso, West Africa [16], and more recently in rural Tanzania (Kaindoa et al. pers. commun.). While more studies are needed to show potential of the approach, it is being promoted as one of the candidates particularly suitable for outdoor use, and for rapid crashing of vector populations. Though studies on *Anopheles* swarms have been minimal in East Africa, it offers a unique opportunity to create complementary new tool that could be used alongside LLINs and IRS to further drive down malaria transmission.

In recent years, progress has been made particularly in identification and characterization of *Anopheles* swarms in Tanzania, raising hopes that this practice could indeed be further explored and potentially targeted to improve control. In a recent study, with the help of community volunteers, Kaindoa et al. identified 216 mosquito swarms three villages in the Kilombero Valley, in south-eastern Tanzania [18]. They further demonstrated, using techniques initially deployed in Burkina Faso [16] that these *Anopheles* swarms can be targeted with help from community members leading to rapid crash in vector populations (Kaindoa et al. pers. commun.). Based on these initial findings, it was postulated that it is essential to explore the different ways that community members would interact with such interventions and what their understandings of mosquito swarms actually are [18]. Improved understanding of such aspects would help fine-tune future interventions that rely on community members to target malaria mosquito swarms. This current study assessed the knowledge and perception of community members regarding presence and role of mosquito swarms in their communities. The study also examined general perceptions of the role of male mosquitoes and role of mosquito swarms in malaria transmission, as well as likelihood of people accepting swarm-targeting interventions for the control of malaria vectors and transmission.

In line with previous research [19–21], the community members who participated in this study had a fairly high awareness of the general role of mosquitoes in malaria transmission. The awareness on role of male mosquitoes in malaria transmission was however relatively low. While all participants believed that malaria was transmitted by only female mosquitoes, most believed that male mosquitoes also fed on blood, but they did not carry malaria parasites as the females do. Male mosquitoes were believed to be responsible for the early-evening bites. Only a few respondents associated swarming with mating, and many did not know that swarms were comprised of just male mosquitoes.

The understanding of what constitutes a mosquito swarm also varied. While majority of the survey respondents reported to have seen mosquito swarms, it is possible that what the respondents reported to have seen were actually not mosquito swarms. Only about two-thirds of the respondents reported to have seen mosquito swarms at or after sunset, calling into question the validity of those that had reported seeing swarms during the day or at night. Similarly, a proportion of participants reported seeing mosquito swarms near light bulbs at night, which clearly indicates that what they were referring to was not mosquito swarms, but simply congregations of insects (possibly including mosquitoes) normally attracted to light bulbs. It is important to understand the current level of knowledge and awareness however, as this will pave way for effective awareness-raising interventions.

The community members perceived the risk mosquito swarms pose to be higher than what experts believe to be the case. The majority believed that mosquitoes were swarming to make plans to look for food (often blood), or to go and attack people thereafter. By this rationale, community members argued that an intervention that would eliminate the mosquito swarms would be necessary and welcomed. This is well explained by the health belief model (HBM) [35], which states that individual’s acceptance of an intervention is shaped by their perceived risk of the problem that the intervention is attempting to fix. The community’s perceptions associated with mosquito swarms were related to their perceptions of malaria risk. While mosquito swarms were not considered an immediate risk, it was widely believed that the swarming mosquitoes would eventually disband, attack people and transmit malaria later in the night. This belief increased the perception of the risk of mosquito swarms and as a result the need for an intervention to eliminate the swarms. While there were indications that the people would be willing to accept and even contribute towards such interventions, proper cost-effectiveness and willingness-to-pay analyses are needed to better understand such potential implication of such aspects in a real intervention implementation.

Public attitude in an intervention such as this is crucial. Swarming mosquitoes are found outdoors, and in a variety of places throughout the community and so interventions that target these mosquitoes can affect all community members, whether they give personal consent or not. It is therefore necessary to ensure that the perceptions and opinions of the community members are understood in order to build meaningful dialogue between the community and the researchers.

## Conclusion

The findings of this study indicate fairly high knowledge about malaria transmission and dynamics of biting risk across seasons. The role of female *Anopheles* mosquitoes in malaria transmission was also very well understood, but there were mixed views on the role of male mosquitoes in malaria transmission. Majority of the community members were aware of presence of mosquito swarms in their communities, but swarms were generally not associated with mosquito mating. Instead, the phenomenon was mostly associated with mosquitoes looking for food or planning to attack people, a belief that increased perception of risk of mosquito swarms. This high perception of risk in turn resulted in willingness to accept and even contribute financially towards interventions that would target swarming mosquitoes. Though the likelihood of actual interventions targeting swarms is still low, such community knowledge will be crucial in future studies of biology of mosquito swarms and possible inclusion of community members in any control efforts.
